# *In situ* flow pair distribution function analysis to probe the assembly–disassembly–organisation–reassembly (ADOR) mechanism of zeolite IPC-2 synthesis†

**DOI:** 10.1039/d1ma00335f

**Published:** 2021-10-13

**Authors:** Samantha E. Russell, Susan E. Henkelis, Simon M. Vornholt, Daniel N. Rainer, Karena W. Chapman, Russell E. Morris

**Affiliations:** School of Chemistry, University of St. Andrews North Haugh St. Andrews Fife KY16 9ST UK rem1@st-andrews.ac.uk; X-ray Science Division, Advanced Photon Source, Argonne National Laboratory Lemont IL 60439 USA

## Abstract

The assembly–disassembly–organisation–reassembly (ADOR) process is an important tool to access zeolite structures that are otherwise unfeasible *via* hydrothermal methods. *In situ* flow pair distribution function (PDF) analysis has been used to probe the mechanism of the disassembly and organisation steps, with the disassembly a rapid step that is often difficult to capture. Zeolite UTL was hydrolysed by 6 M hydrochloric acid, with PDF measurements used to monitor framework alterations as the reaction proceeded. The resulting disassembly mechanism shows an initial rapid removal of germanium from the germanium-rich double 4 rings (d4r), followed by silicon rearrangement and gradual silanol condensation to form IPC-2P.

## Abstract 2

A four step mechanism for the disassembly and organisation stages of the ADOR process was determined using *in situ* flow pair distribution function.

## Graphical abstract

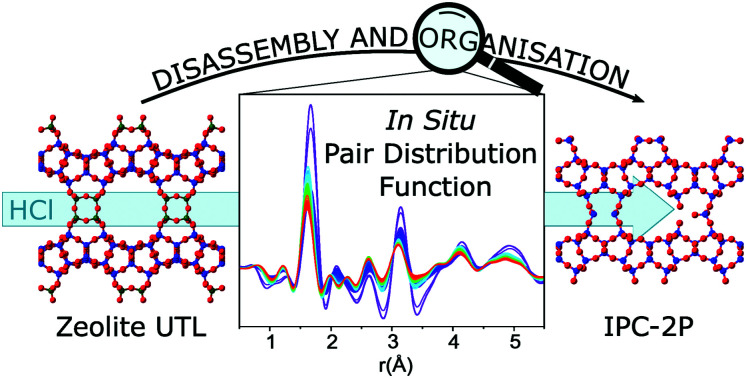

## Introduction

Zeolites are an important class of porous materials that are connected through TO_4_-tetrahedra, where T is most commonly silicon, but can also be other elements such as aluminium and germanium. Such zeolites are traditionally synthesised through hydrothermal methods,^1,2^ though in recent times, a different method has been developed: assembly–disassembly–organization–reassembly (ADOR). This four-step process allows for the formation of new zeolites that are typically unfeasible through traditional synthesis.^3^ The ADOR method begins with the assembly (A) of a parent germanosilicate (zeolite) where the germanium is preferentially located within the double 4 rings (d4r) of the 3D framework. These labile germanium–oxygen linkages introduce a controlled weakness into the framework, and as such can then be disassembled (D) under aqueous conditions to produce a Si-rich layered material.^4^ These siliceous layers can then be further organised (O) and reassembled (R) to form new zeolite frameworks that differ from the initial structure.

**Fig. 1:**
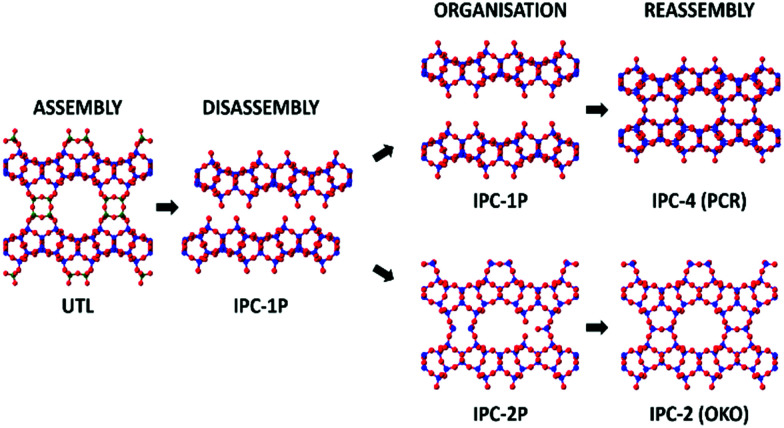
An overview of two different routes in the ADOR process showing the assembly, disassembly, organisation, and reassembly of the parent zeolite UTL to final products IPC-4 and IPC-2. Blue atoms represent the T-atoms within the layers, which are predominantly silicon, dark green represents T-atoms of the double 4 rings (d4r) that are a combination of silicon and germanium, where the ratio is dependent upon synthesis conditions, and red are oxygen atoms.

A range of parent zeolites have proved to be suitable candidates for the ADOR process, including IM-17 (UOV), ITQ-22 (IWW) and SAZ-1 (*CTH), all of which contain the essential Ge-rich d4r unit that permits the controlled disassembly.^5–8^ One of the most interesting parent zeolites to date is the UTL framework, which has so far been shown to produce six different high silica zeolite products *via* the ADOR method; namely IPC-2, 4, 6, 7, 9 and 10.^9–11^ A schematic of the ADOR process can be seen in Fig. 1, highlighting two different pathways from parent zeolite UTL; one to IPC-4 (PCR) and the other to IPC-2 (OKO).

A range of techniques have been employed to study the ADOR process, including, but not limited to, powder X-ray diffraction (PXRD) and solid-state NMR.^12–15^ The rapid disassembly of the framework, however, results in a loss of some long range order, making it challenging to follow accurately *via* traditional X-ray diffraction characterisation techniques. Since total scattering experiments consider both the Bragg and diffuse components of scattering, it is extremely useful for probing materials where disorder is present.^16^ Such experiments result in the acquisition of the pair distribution function (PDF), which provides a measure of the interatomic distances within a structure, regardless of crystallinity. The use of PDF can therefore provide an understanding of the atomic level changes occurring throughout the ADOR process.^17,18^

PDF of the ADOR process has previously been used with great success to determine the structure of IPC-1P, the disordered intermediate of disassembled UTL.^19^ Refinement of a density functional theory (DFT) modelled structure against IPC-1P PDF data helped to elucidate its structure and confirmed that the layers from the UTL structure remain intact upon disassembly. The structural stages of the disassembly/organisation steps under different hydrolysis conditions have also been probed using *in situ* PDF in a closed small volume system.^20^ The study concluded that water as the hydrolysing media results in a slower hydrolysis than aqueous solutions of 6 M and 12 M hydrochloric acid and that the final disassembled structure for the water hydrolysis is IPC-1P. Contrary to this, the 6 M and 12 M hydrochloric acid data both showed an induction period, followed by rearrangement, resulting in final structures of IPC-7P and IPC-2P, respectively. It was concluded that the high [H^+^] was a crucial factor in determining the rate and final product of the disassembly/organisation.

Due to the closed nature of the system used in the previous *in situ* PDF work, products formed during the d4r hydrolysis process remained within the reaction cell thus contributing to the scattering data, and therefore to the final PDF. To obtain a better understanding of the bulk framework changes, we hereby present for the first time an investigation in to the ADOR process in a flow system, therefore allowing for the removal of any structural debris. *In situ* PDF measurements were recorded to monitor the interatomic distances during hydrolysis, revealing any structural changes occurring. This allowed for a time resolved tracking and identification of the order that the key steps occur during the ADOR process under a flow system. In addition, it is clear from previous work that small volume batch synthesis, including that for the previous *in situ* PDF study, do not replicate the conditions of larger scale synthesis adequately, because the lower volumes of solvent do not provide as strong a driving force for the removal of hydrolysis debris from between the layers.^12,15^ The flow-based *in situ* technique reported here allows, for the first time, the continuous removal of structural debris partnered with the steady supply of fresh solution, a very different set of conditions than any work undertaken previously.

## Experimental

### Materials and methods

UTL was synthesised following a previously reported procedure that uses (6*R*,10*S*)-6,10-dimethyl-5-azoniaspiro[4,5]decane hydroxide as the structure directing agent (SDA).^20^ The resulting UTL was calcined at 575 °C for 7 h, with a heating ramp of 1 °C min^−1^, to remove the organic SDA. The final Si/Ge ratio of 6.2 was determined by energy-dispersive X-ray spectroscopy, a ratio that results in an average d4r unit composition of [5Ge,3Si].^3^

### Total scattering measurements

**Fig. 2:**
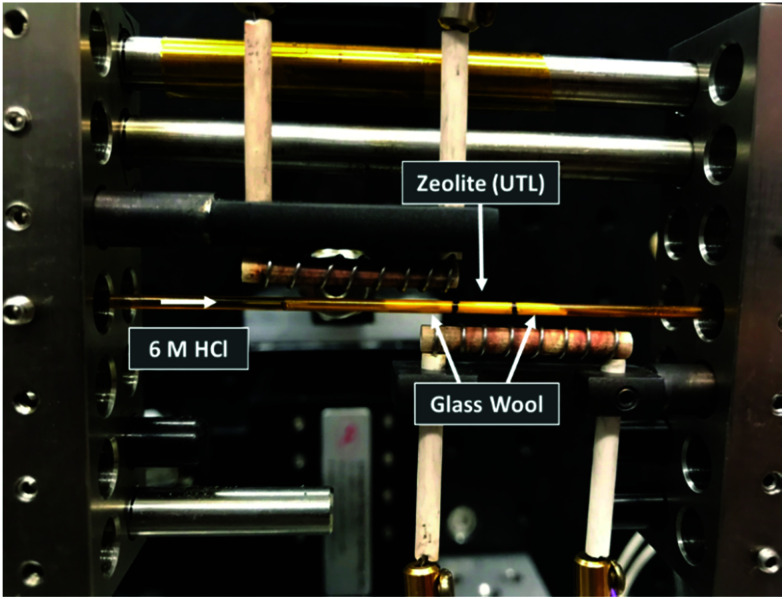
The experimental set-up of the *in situ* flow hydrolysis of zeolite UTL. The zeolite was held in place between two plugs of glass wool, with the sample region denoted by the black lines, and the acid pumped through the sample.

Total scattering measurements were performed at beamline 11-ID-B at the Advanced Photon Source (APS) using high energy X-rays (58 keV, *λ* = 0.2113 Å), equipped with an amorphous Si-based PerkinElmer detector. The UTL was placed in a Kapton capillary, with 5 mm of sample packed in between glass wool to hold the powder in place, as shown in Fig. 2. Hydrochloric acid (6 M) was pumped into the capillary at a constant rate of 0.1 mL min^−1^ and data collected at time intervals of 10 s for the first 5 min, followed by 65 s intervals for 1 h and lastly 5 min intervals for 9 h and 25 min. The data were recorded for 10 h and 30 min in total. The hydrolysis was performed at 100 °C, achieved by the use of heating coils that can be observed in Fig. 2. The collected data were reduced using Fit2d and the total scattering data, *S*(*Q*), were transformed to the *G*(*r*) data *via* Fourier transformation.^21^ This was performed using PDFgetX2 with a chemical composition of SiO_2_.^22^ Although the true chemical composition of the sample will vary slightly throughout the hydrolysis process, a single composition of SiO_2_ was applied to all data sets. This was due to the inability to know specific compositions at each stage of the process and therefore preventing exact compositions from being applied to each data set. However, we do know that Ge-removal is rapid and the resulting hydrolysis products are almost purely siliceous, therefore a composition of SiO_2_ is reasonable for the bulk of the process. Nevertheless, this single composition may still result in some systematic errors in the PDF intensities. The *S*(*Q*) data was collected out to 22 Å^−1^, resulting in *G*(*r*) data measuring out to 30 Å. Acquired data were corrected for background and inelastic scattering using Fit2d, where background data were recorded using an acid-filled Kapton capillary.

## Results and discussion

**Fig. 3:**
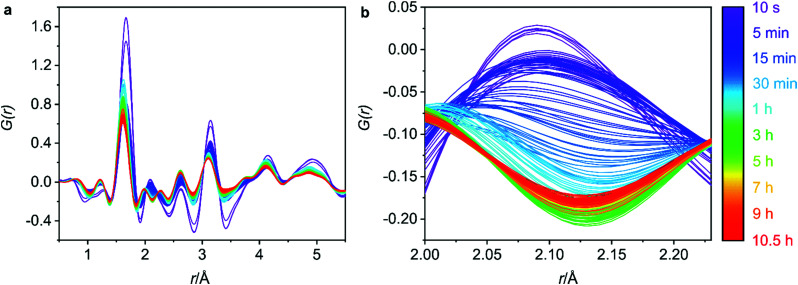
(a) The 0.5–5.5 Å region of the 6 M hydrochloric acid hydrolysis of UTL. (b) The Ge–Cl interatomic distance that decreases over time as germanium is removed from the UTL framework. The Ge–Cl product is removed due to the flow nature of the experiment.

Phase purity of the bulk materials was ensured *via* PXRD methods prior to subjecting the zeolite UTL sample to hydrolysis. Furthermore, SEM (scanning electron microscopy) micrographs were captured and show decisively one morphology, both the PXRD and the SEM micrographs are presented in Fig. S1 (ESI†). The *G*(*r*) data for the 0.5–5.5 Å region is displayed in Fig. 3a and represents the key interatomic distances before ring overlap and secondary atomic distances are observed. The full range of the *G*(*r*) data, from 0–30 Å, is presented in Fig. S2 (ESI†).

Firstly, it is important to identify the final structure obtained, as this will help to connect the processes that are occurring throughout the PDF measurements with the final product. Converting the total scattering data collected in *Q* to 2*θ* with a Cu Kα wavelength (1.54 Å), provided the 2*θ* peak position of the low-angle diffraction peak. This peak corresponds to the *d*_200_ interlayer distance and is commonly followed in ADOR reactions to observe the distance between the silica-rich layers and therefore identify the ADOR product present.^23^ The 2*θ* position (Fig. S3, ESI†) around 7.35° indicates that the product at the end of the data collection is IPC-2P, the partially connected intermediate of IPC-2, with a typical peak position around 7.5°.^12^ The proposed final product of IPC-2P is further supported by in-house PXRD data of the recovered hydrolysis sample (from the APS) and the following calcination to produce IPC-2 (Fig. S4, ESI†). A subsequent Le Bail fit of the calcined powder pattern confirmed the appropriate unit cell for IPC-2 (Fig. S5, ESI†), reinforcing the identification of IPC-2 as the calcined final material.

Secondly, it is important to determine what stage of the process we are capturing with the first data set, as the initial hydrolysis is often rapid and therefore difficult to observe. Considering the *G*(*r*) data presented in Fig. 3a, the lack of peak at 4.5 Å, a peak that represents the diagonal T–T distance of the d4r of UTL,^20^ indicates that hydrolysis of the starting UTL has already commenced. It is likely that due to the use of acid in the set-up shown in Fig. 2, the acid vapour has reached the sample prior to the start of data acquisition, initiating the d4r hydrolysis. This type of acid vapour-phase hydrolysis has previously been observed for zeolite UTL, as well as other frameworks.^6^ A test run of the set-up using water shows the presence of this diagonal d4r distance in the first data set, Fig. S6 (ESI†), that rapidly disappears as the hydrolysis commences, supporting that this peak would have been present had the acid vapour not initiated the d4r hydrolysis.

**Fig. 4:**
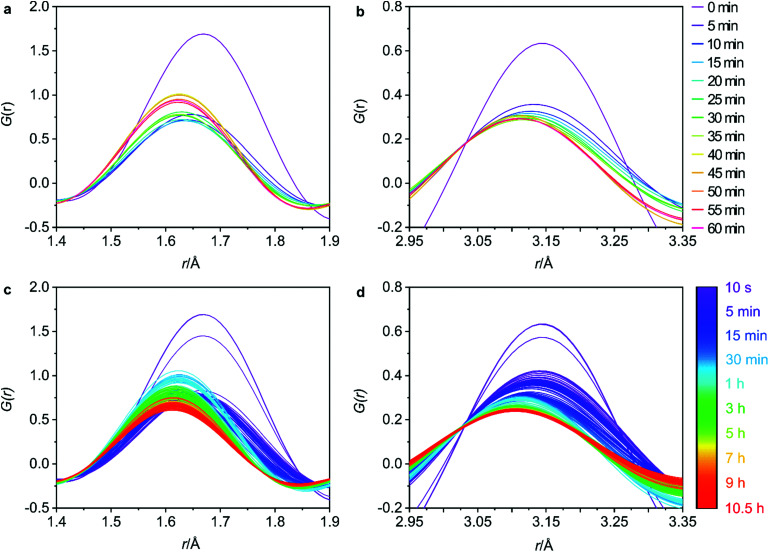
The changes in the T–O (a) and T–T (b) interatomic distances at regular 5 minute intervals for the first hour and the full data sets for the T–O (c) and T–T (d) distances to observe the overall trends.

Although the hydrolysis may have already commenced, germanium atoms are still present in the system, indicating that the rapid hydrolysis step is still occurring. This is confirmed by the peak around 2.1 Å that corresponds to the Ge–Cl bond distance, a bond formation that occurs as the d4r germanium is hydrolysed in the presence of the hydrochloric acid. The initial data set shows a peak corresponding to this bond length (2.1 Å), Fig. 3b, indicating ongoing germanium removal. Further to this, there is an observed decrease in the length of the T–O and T–T distances, Fig. 4a and b, which are later discussed in further detail, but indicates ongoing Ge-removal from the framework. This simultaneous loss of framework germanium and observed presence of Ge–Cl indicates that we are actively capturing the loss of germanium from the framework in the presence of the hydrochloric acid. As the experiment was run under flow conditions, the Ge–Cl species that are formed are then removed from the system with the flowing acid, this is shown by the reduction of this peak as the reaction proceeds. Partial PDFs were calculated for UTL, Fig. S7 (ESI†), to exclude the possibility of this peak relating to any other UTL framework distance.

The Ge–Cl formation allows for a relatively easy identification of the Ge-removal. The following steps of the hydrolysis however, are much more challenging to determine. In order to identify the key structural changes of the UTL as it undergoes hydrolysis, the *G*(*r*) data was plotted over regular time intervals of 5 min to help follow significant changes over time. There are three main interatomic distances in the zeolite structure that can be followed; T–O (where T is silicon or germanium), T–T and O–O. Monitoring these distances provides information on the disassembly and organisation mechanism as the UTL undergoes hydrolysis by the aqueous acid.

The 5 min interval plots indicate significant changes to the peak positions and intensities occurring within the first hour of data collection for both the T–O (Fig. 4a) and the T–T (Fig. 4b) distances. Firstly, considering the peak positions, in the first 5 min there is a decrease in both the T–O and T–T distances. This can also be observed in Fig. S8b and d (ESI†) where the peak positions for T–O and T–T respectively, have been plotted against reaction time for both distances. This shift would be consistent with a loss of germanium from the d4r of UTL as the larger size of germanium atoms results in longer Ge–O bonds than Si–O bonds, and similarly interatomic distances between T–T atoms will likely decrease in the order of Ge–Ge > Ge–Si > Si–Si. Therefore, the loss of germanium would result in shortening of both the average T–T and T–O distances. This shortening occurs within the first 0–5 min of the hydrolysis, suggesting that the majority of the germanium is removed within this time frame. There is some remaining gradual movement in both peak positions over time, as best highlighted in Fig. S8b and d (ESI†). This slight shift likely indicates the removal of any residual germanium that could be present within the layers in small amounts.

Considering the peak intensities, the first key change occurs between 0–5 min where there is a decrease in the intensity for both distances, Fig. 4a and b, which supports the germanium removal, as the total number of T–O and T–T distances will be reduced. After this initial sudden decrease in intensity, there remains a continuous gradual movement for both peaks, especially for T–O as shown in Fig. 4c where the intensity is still decreasing towards the end of the 10.5 h data collection. As has been observed previously, under certain conditions, silicon lost from the d4r during the hydrolysis can intercalate between the layers to form new single 4 ring (s4r) connections.^9^ Further to this, it was found that silicon from the Si-rich layers could also rearrange and intercalate between the layers, showing just how dynamic this system can be.^13^ Considering Fig. S3 (ESI†) where we observe the *d*_200_ interlayer distance peak, it is apparent that under these conditions, the material does not reach IPC-1P, the fully disassembled ADOR intermediate. This common intermediate is often a stepping-stone during an induction period prior to further rearrangement to materials such as IPC-2P or IPC-7P. This induction period was observed during the previous PDF studies with 6 M HCl, but the movement observed from the PDF data presented here indicates that we have completely by-passed IPC-1P, never reaching the fully layered structure.^20^ Instead, after the initial loss of germanium, we observe a rearrangement of silicon within the system. This observation is further supported by previous solid-state NMR work that studied the hydrolysis of UTL with 6 M HCl under low volume conditions.^15^ As was also observed here, the NMR findings showed no evidence of the intermediate IPC-1P and instead showed gradual rearrangement to a final resulting structure of IPC-2P. The intensities of the Q^4^ (Si(OSi)_4_) and Q^3^ (Si(OSi)_3_(OH)) species in the hydrolysed samples were monitored, with the variation in Q^4^/Q^3^ ratio revealing significant local changes occurring for a considerable time period after the initial hydrolysis. The similarity of the steps observed between these two studies, suggests that the *in situ* flow disassembly and organisation process follows more closely to the low volume hydrolysis, as opposed to the previous large volume work. This shows the strong influence that the different experimental conditions have on the mechanism of the ADOR process.

Although not as extreme as the first few minutes, the full data sets for T–O and T–T, Fig. 4c and d respectively, show that there is still a lot of movement throughout the whole 10.5 h. The T–O intensity shows a constant decrease from around 45 min until the final data set, thus indicating a loss of T–O distances. This observed decrease paired with the end product of IPC-2P suggests that the silanol groups are slowly condensing over time to begin to connect up the s4r linkages.

**Fig. 5:**
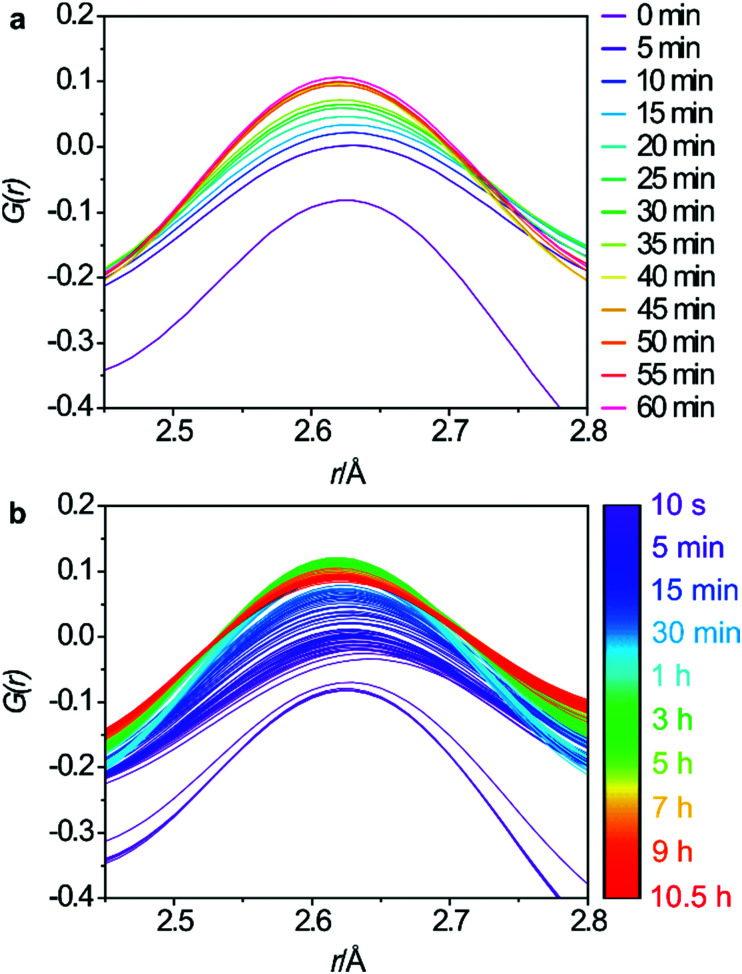
Regular 5 minute intervals of the O–O interatomic distances for the first hour (a) and the full data set (b).

The changes to the third key peak, representing the O–O interatomic distances, are shown in Fig. 5a and b. There is a rapid increase in peak intensity within the first 5 min which then becomes a gradual increase until around 45 min where the intensity stabilises. This supports the Si-rearrangement during this period. However, it is likely that the O–O distances in the PDF are not the best indicator of the mechanism as it is difficult to separate the framework O–O distances from those in the aqueous phase that are present both within the pores of the zeolite and around the particles of solid.

**Fig. 6:**
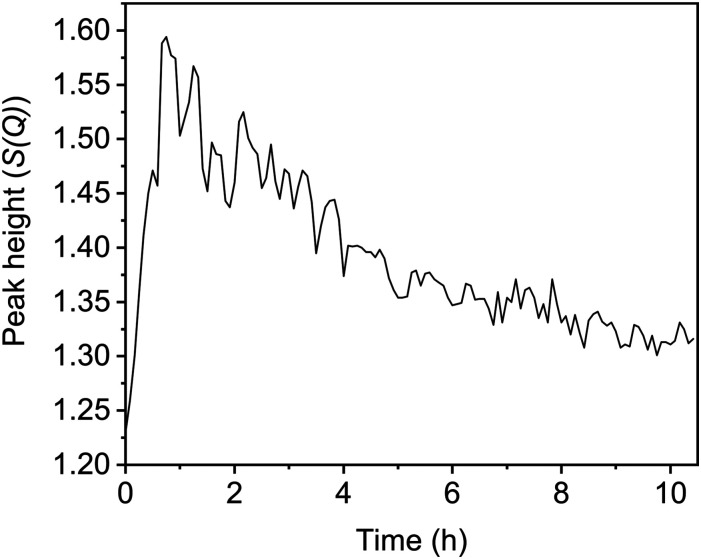
The change in the peak height of the *S*(*Q*) first sharp diffraction peak (FSDP) over the course of the reaction.

In addition to the *G*(*r*) data, the total scattering data (*S*(*Q*)) can highlight periods of significant change within the UTL structure. One key peak is the first sharp diffraction peak (FSDP), where changes to the position and intensity of the peak signify changes in the composition or density of the zeolite structure.^24–26^ Furthermore, as previously mentioned, this peak relates to the interlayer spacing and therefore has strong correlations to the linkages between the layers, or lack thereof. The full *S*(*Q*) data with the FSDP highlighted is presented in Fig. S9 (ESI†). The peak height of the FSDP, Fig. 6, shows an initial rapid increase occurring during the first 45 min that then slowly decreases again over time before starting to plateau around 10 h. This time period aligns with the timings of the peak shifts in the *G*(*r*) data, with peak intensities shifting during the first 40 min before steadily decreasing over time. The initial sudden increase is supportive of a rapid Ge-removal and commencement of the Si-rearrangement, with the continuous decrease that follows supporting the idea of gradual silanol condensation.

**Fig. 7:**
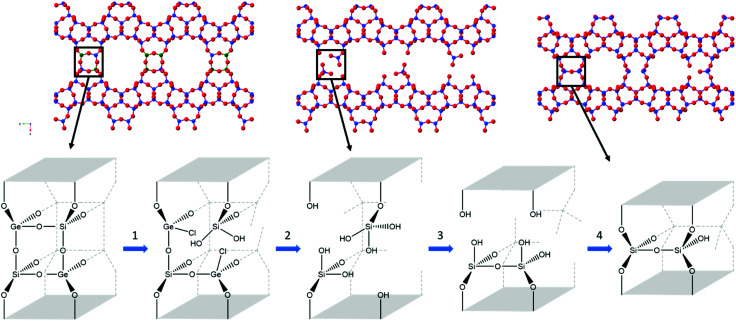
The four steps observed in the hydrolysis of UTL with 6 M hydrochloric acid, with the crystallographic representation (top) and the d4r highlighting key bond breakages and formation (bottom). In the crystal structure, blue represents silicon, green for germanium and red for oxygen. The four key steps show the attack of chloride at the germanium d4r atoms (1), removal of germanium (2), silicon rearrangement (3) and condensation of silanol groups (4).

The data obtained indicates a series of processes occurring throughout the disassembly and organisation steps at varying times. Overall, there are four key stages occurring during the 6 M HCl hydrolysis, as represented in Fig. 7. Firstly, the chloride from the acid attacks the germanium atom within the d4r, a process that occurs too rapidly for the experimental set-up to capture. Secondly, after the chloride attack, the germanium is removed from the d4r units. This stage has been captured, as represented by the decreasing presence of a Ge–Cl peak and the shortening of average distances for both T–O and T–T. The third step is the rearrangement of the silicon that is observed by the movement of intensity for the T–O and T–T peaks and the lack of induction period, a prominent step that was observed in the previous PDF studies.^20^ Lastly is the condensation of the silanol groups, a gradual process that is represented by the slowly decreasing intensity of the T–O peak and the decreasing peak height of the FSDP. This four-step hydrolysis results in a final layered material of IPC-2P.

## Conclusions

The flow system has successfully allowed an *in situ* PDF study of the ADOR process. The use of the flow system enabled the removal of any structural debris, therefore placing the focus on the UTL framework changes and provided an insight into the disassembly and organisation mechanism. A four-step mechanism has been proposed that is supported by the observed structural changes from both the *G*(*r*) and the *S*(*Q*) data. This shows an initial rapid attack at the germanium from the acid, resulting in the subsequent loss of the d4r germanium. This is followed by silicon rearrangement and slow silanol condensation to produce IPC-2P. To better capture the initial rapid hydrolysis, future work should look at changes to the experimental set-up to try prevent the acid vapour from starting the hydrolysis prior to data collection. One solution could include not having the acid as close to the sample before starting the experiment, therefore preventing any vapour from being able to reach the zeolite. A second option would be to try avoid the overlapping of the acid and the heating coils prior to the experiment, as this will increase the acid vapour present.

The *in situ* PDF data collected in the experiments reported here is important for several reasons. As we gain more and more detail of the mechanism by which the ADOR process works, we are in a better position to both understand and to manipulate the process to provide improvements. In particular, our understanding of the difference between the behaviour of germanium and silicon in different systems when challenged by the hydrolysis solution offers us the possibility of developing systems and protocols that require the minimum amount of germanium in the solid to more efficiently complete the ADOR process. It also adds important fundamental information that helps us understand the reactivity of zeolites with aqueous solutions: an area that is becoming ever more important as zeolites come under greater scrutiny for applications such as the refining of biomass that takes place under aqueous conditions.^27,28^

## Conflicts of interest

There are no conflicts to declare.

## Supplementary Material

MA-002-D1MA00335F-s001
