# Supplementary material for: In situ flow pair distribution function analysis to probe the assembly–disassembly–organisation–reassembly (ADOR) mechanism of zeolite IPC-2 synthesis
Source: Mater Adv. 2021 Oct 13;2(24):7949–55. doi: 10.1039/d1ma00335f (PMC8666841; doi:10.1039/d1ma00335f)
Supplement: MA-002-D1MA00335F-s001 [file MA-002-D1MA00335F-s001.pdf]

Electronic Supplementary Material (ESI) for Materials Advances.
This journal is © The Royal Society of Chemistry 2021

In Situ Flow Pair Distribution Function Analysis to Probe the Assembly-Disassembly-Organisation-Reassembly (ADOR) Mechanism of Zeolite IPC-2 Synthesis Supplementary Information

Samantha E. Russell, Susan E. Henkelis, Simon M. Vornholt, Daniel N. Rainer, Karena W. Chapman and Russell E. Morris

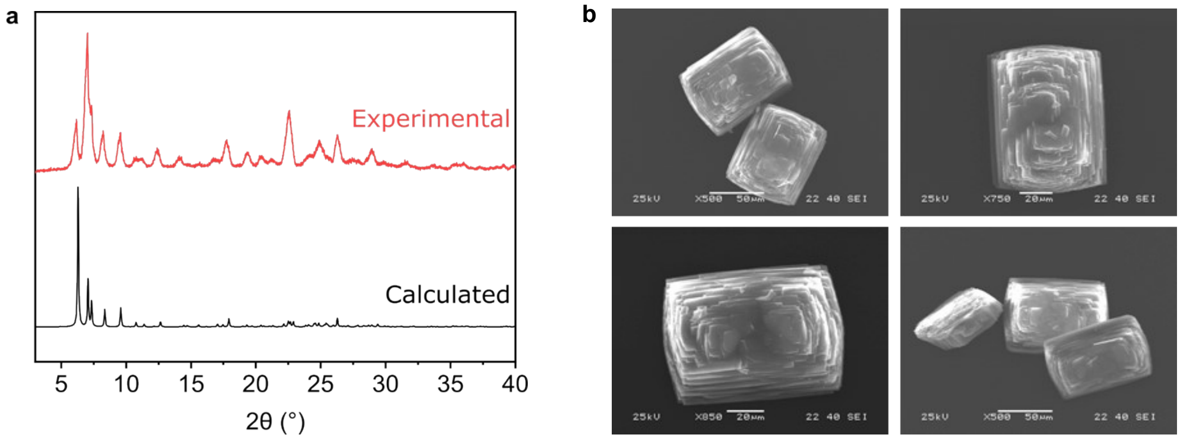

Figure S1. PXRD (a) of the starting **UTL** (red) compared to the calculated **UTL** PXRD (black) from the IZA database.[1] SEM micrographs (b) of the starting **UTL** showing the classic tombstone shape of the **UTL** crystals with no other phases present.

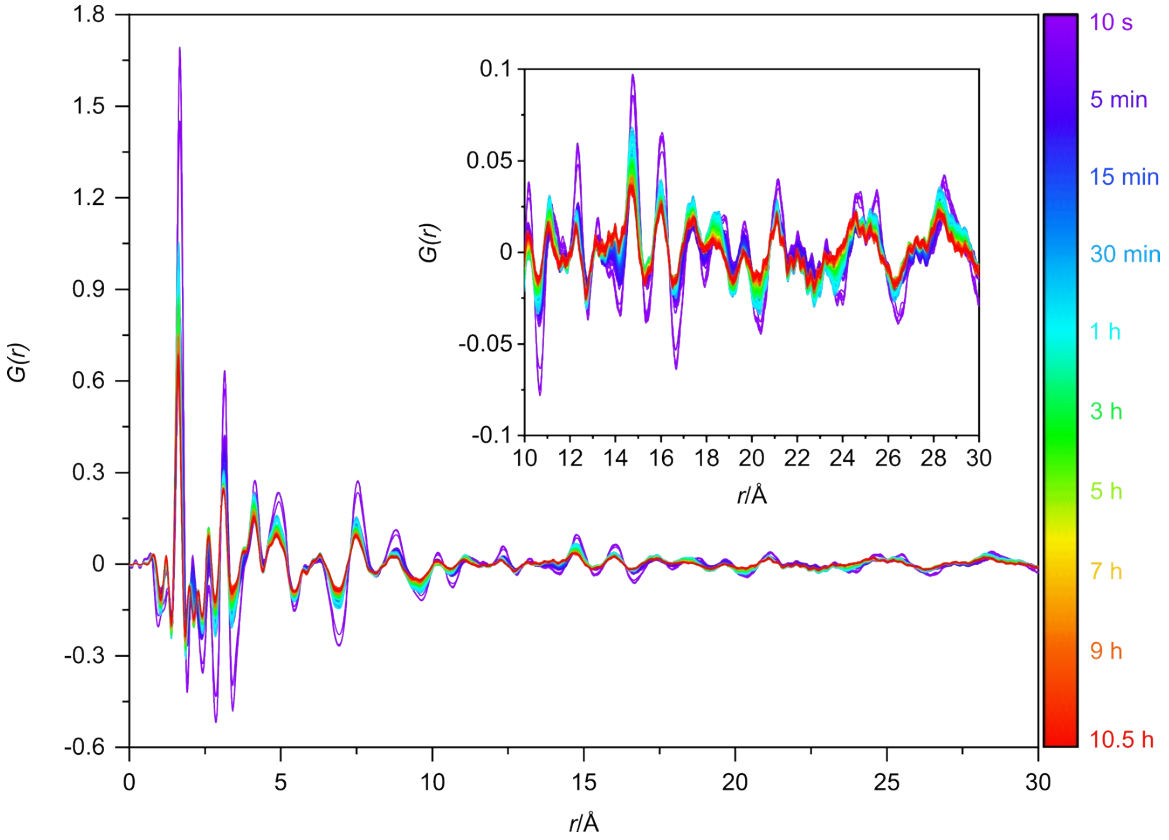

Figure S2. The full range of PDF (*G(r)*) data from 0 to 30 Å, with the 10 to 30 Å region zoomed to show any changes in the longer interatomic distances.

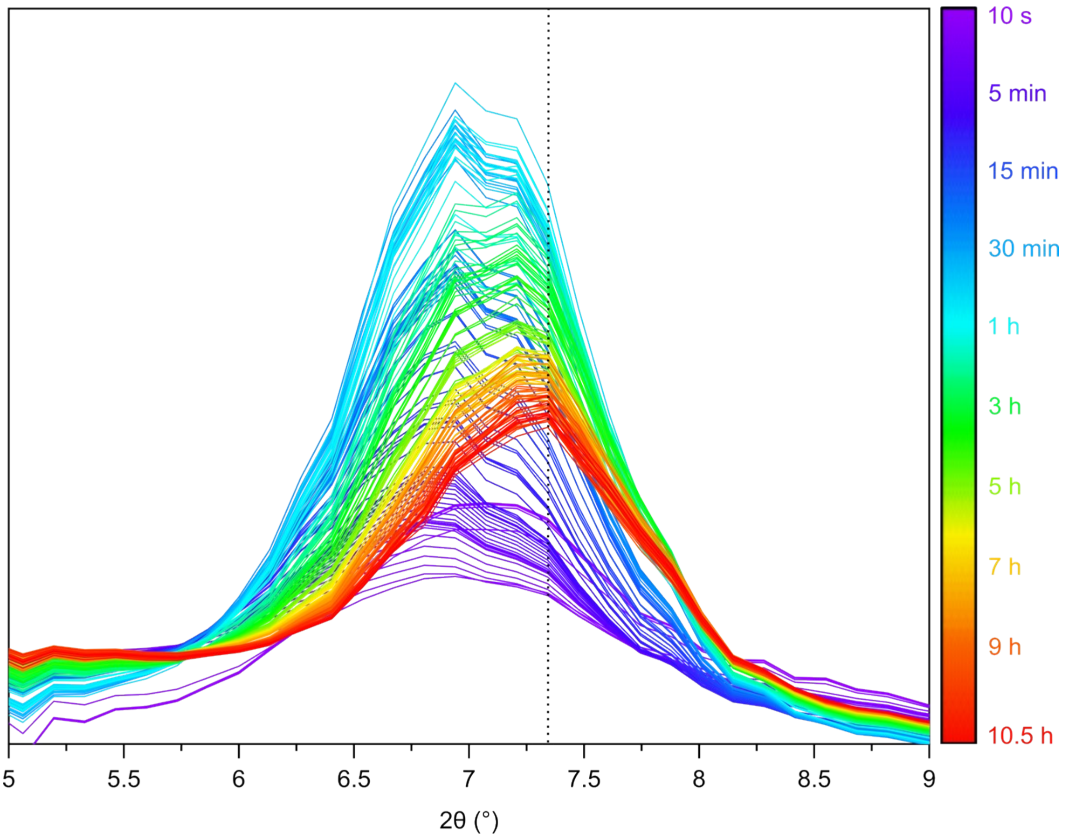

Figure S3. CuK_{α} (1.54 Å) 2θ values of the total scattering data collected in Q to observe the position of the d_{200} interlayer distance peak that is a key indicator of the ADOR product. The dashed line shows the d_{200} peak around 7.35° at the end of data collection.

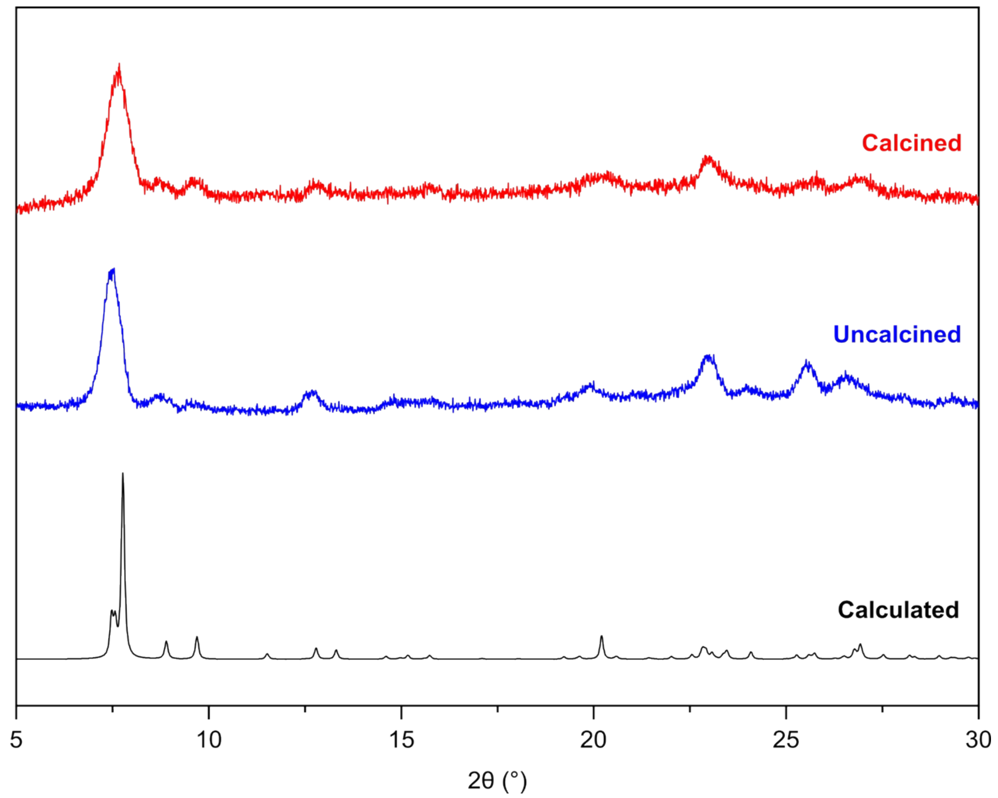

Figure S4. Comparison of the *ex situ* PXRDs for the final structure. Calculated IPC-2 (black) from the IZA database,[1] the uncalcined final product (blue) and the calcined final product (red).

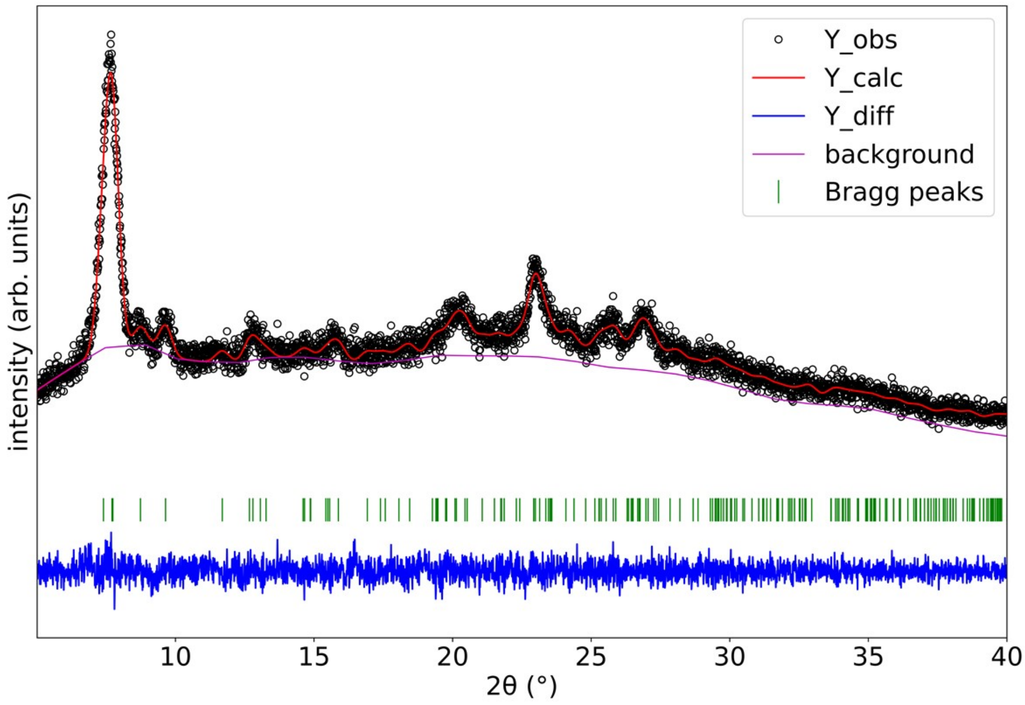

Figure S5. The Le Bail fit of the calcined final product in the C2/m space group, showing the experimental observed data (black circles), the calculated pattern (red), the difference (blue), the background (purple) and the Bragg peaks (green).

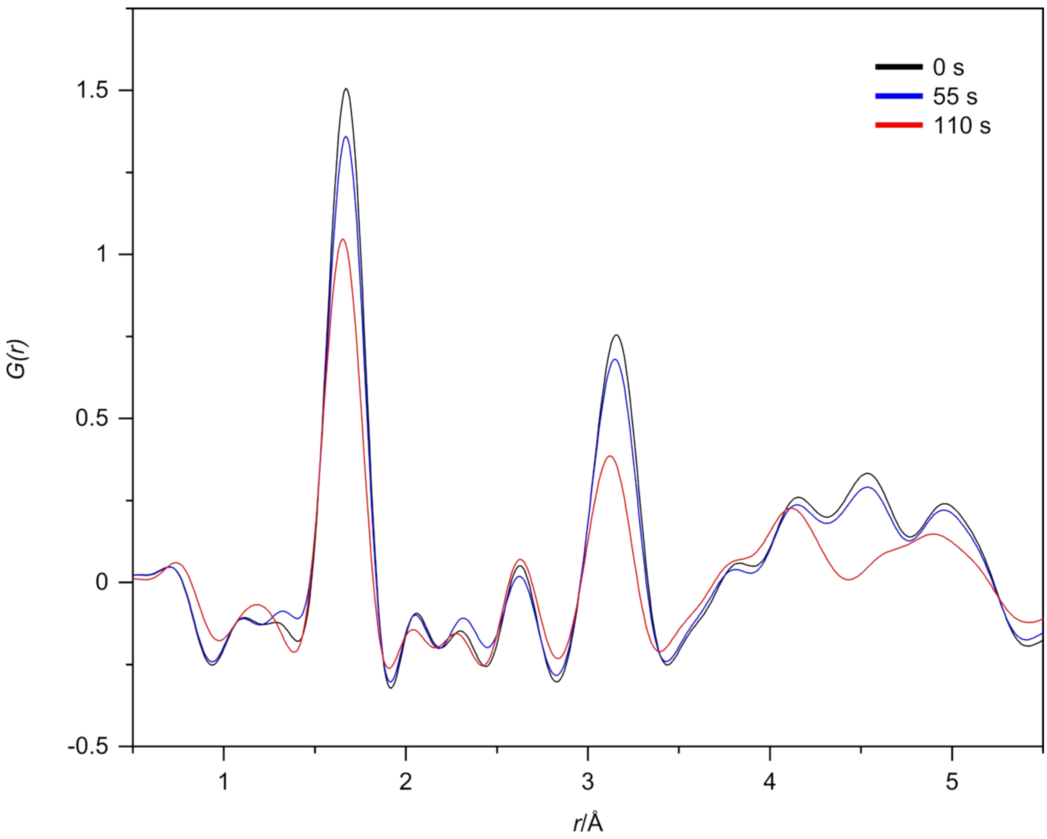

Figure S6. An *in situ* test run using water to hydrolyse the **UTL**. The first three PDFs recorded at 0 s (blue), 55 s (red) and 110 s (black) are presented, showing a notable change by 110 seconds. The key diagonal T–T distance of the d4r of **UTL** is observed at 4.5 Å and has completely disappeared after 110 s. This data supports the claim that the acid vapour had already started the hydrolysis of **UTL** in the case of the 6 M HCl acid, as we see the loss of the T–T distance from the first data collection. It also highlights how rapidly this diagonal T–T distance is disrupted once hydrolysis commences.

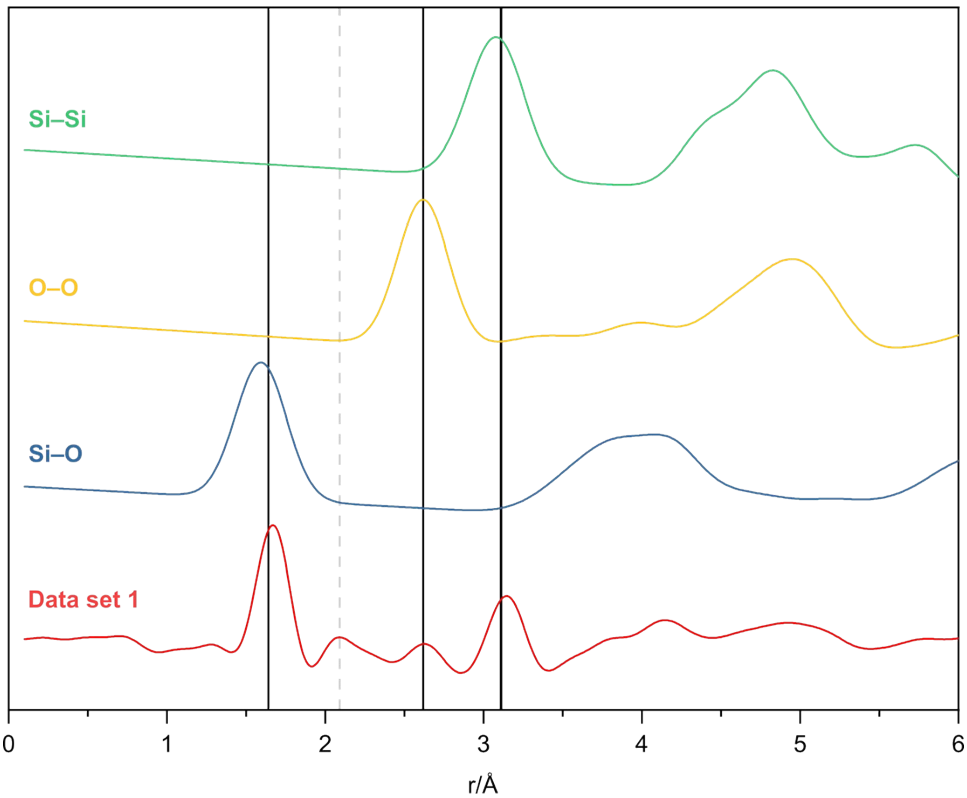

Figure S7. Data set number 1 (red), with the **UTL** partial PDFs for Si–O (blue), O–O (yellow) and Si–Si (green), with the proposed Ge-Cl distance highlighted by the grey dashed line. The lack of any other framework distances corresponding to this peak supports the proposed formation of Ge–Cl bonds in the system.

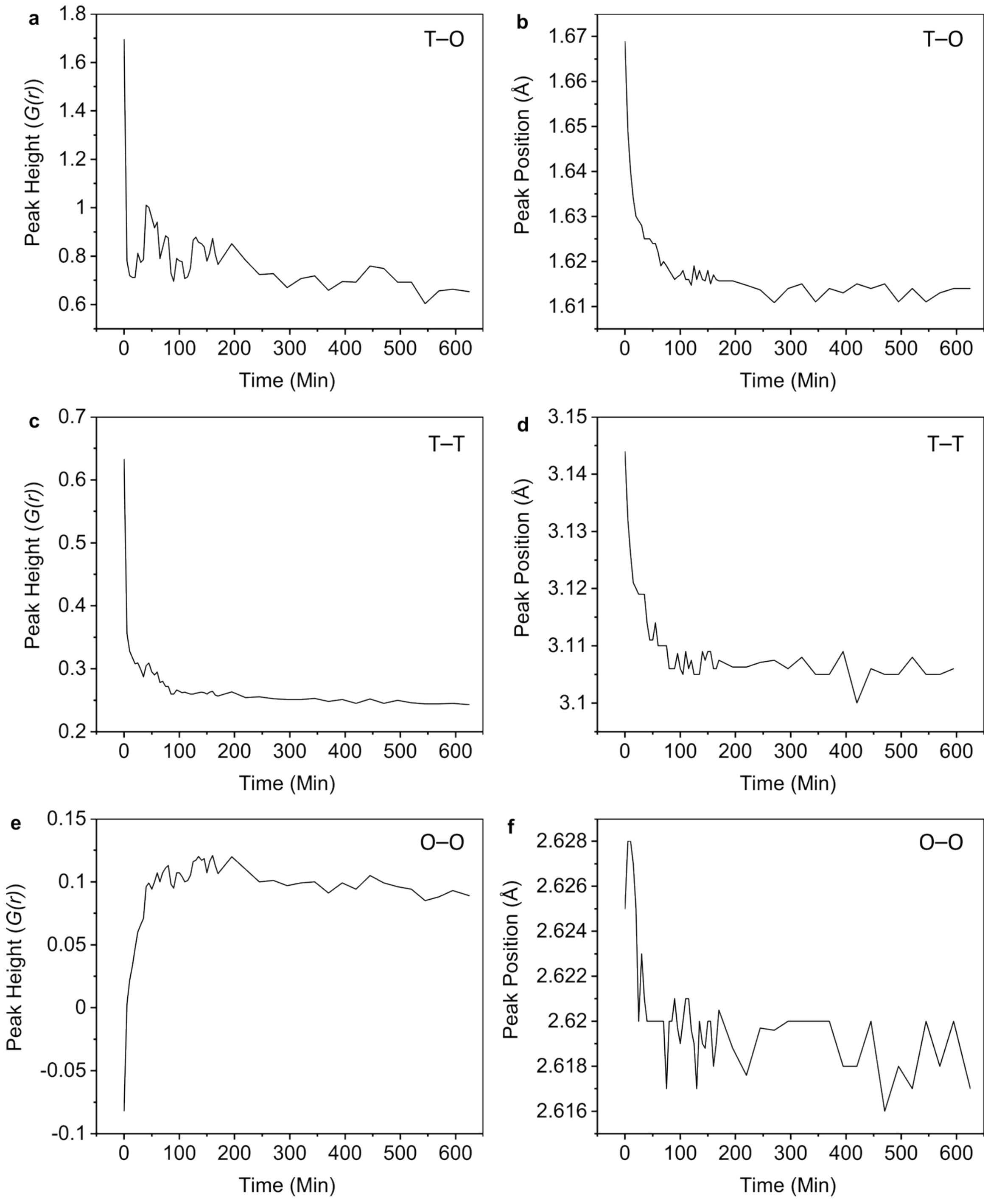

Figure S8. The change in peak height and position of the *G(r)* peaks for T–O (a, b), T–T (c, d) and O–O (e, f) during the hydrolysis.

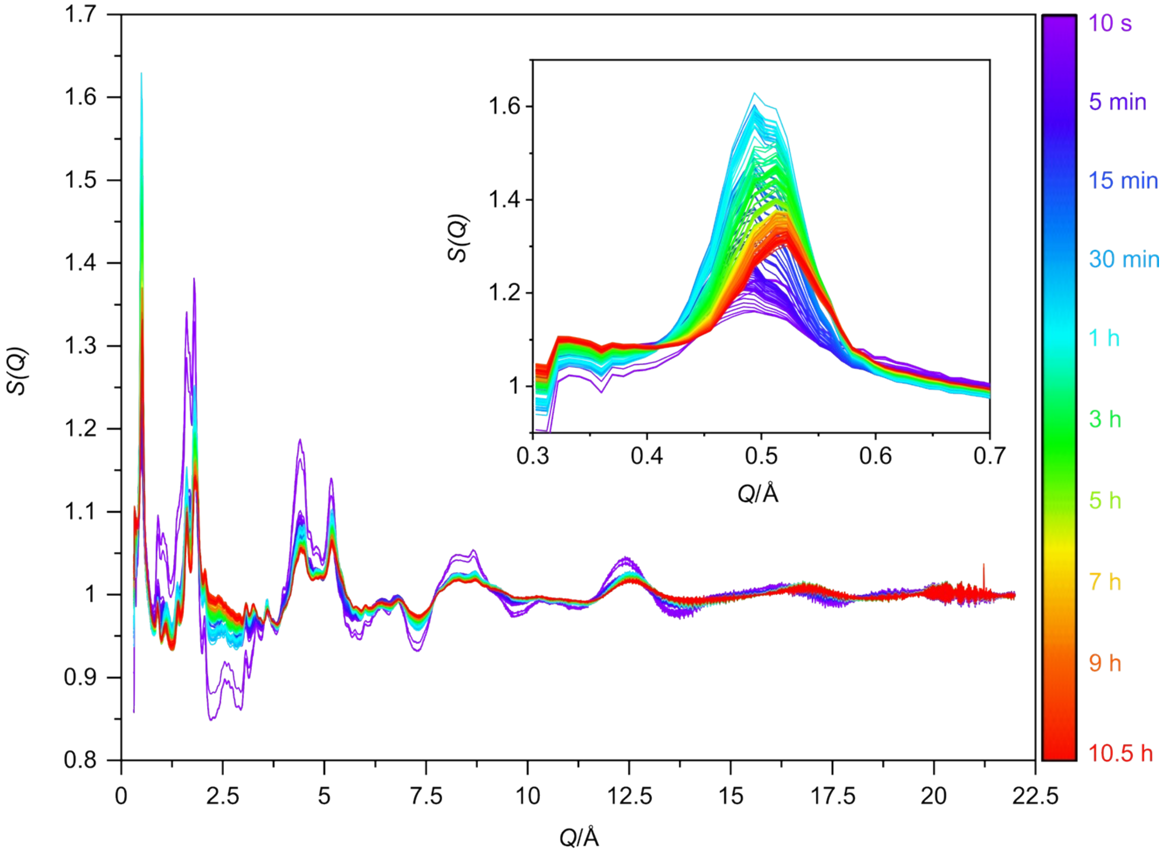

Figure S9. The full range of *S(Q)* data from 0 – 22 Å^{-1}, with the first sharp diffraction peak (FSDP) highlighted in the zoomed region between 0.3 – 0.7 Å^{-1}.

1. Ch. Baerlocher and L.B. McCusker. Database of Zeolite Structures. Available at: http://www.iza-structure.org/databases/. (Accessed Jan 2021)
